# Association of perinatal factors with neurodevelopmental referrals in a population-based cohort study in Japan

**DOI:** 10.1038/s41598-024-54167-w

**Published:** 2024-02-12

**Authors:** Yuki Kyono, Masahiro Nishiyama, Aoi Kawamura, Shizuka Oikawa, Shoichi Tokumoto, Hiroshi Yamaguchi, Kazumi Tomioka, Kandai Nozu, Hiroki Mishina, Hiroaki Nagase

**Affiliations:** 1 Department of Pediatrics, Kobe University Graduate School of Medicine, 7-5-2, Kusunoki-Cho, Chuo-Ku, Kobe, Hyogo 650-0017 Japan; 2 Department of Neurology, Hyogo Prefectural Kobe Children’s Hospital, Kobe, Japan; 3 Kobe City Child and Family Bureau, Kobe, Japan

**Keywords:** Paediatric research, Paediatric research

## Abstract

Although the causes of neurodevelopmental disorders remain unknown, several environmental risk factors have attracted considerable attention. We conducted a retrospective, longitudinal, population-based cohort study using data from infant health examinations of children born to mothers with pregnancies between April 1, 2014 and March 31, 2016 in Kobe City to identify the perinatal factors associated with neurodevelopmental referrals in 3-year-old children. There were 15,223 and 1283 children in the normal and referral groups, respectively. Neurodevelopmental referrals at the health checkup for 3-year-old children were significantly associated with the lack of social support during pregnancy (adjusted odds ratio [aOR] 1.99, 99% CI 1.14–3.45, p = 0.001), history of psychiatric consultation (aOR 1.56, 99% CI 1.10–2.22, p = 0.001), no social assistance post-delivery (aOR 1.49, 99% CI 1.03–2.16, p = 0.006), Edinburgh Post-natal Depression Scale (EPDS) score ≥ 9 (aOR 1.36, 99% CI 1.01–1.84, p = 0.008), infant gender (male) (aOR 2.51, 99% CI 2.05–3.06, p < 0.001), and cesarean delivery (aOR 1.39, 99% CI 1.11–1.75, p < 0.001). In conclusion, this exploratory study in the general Japanese population identified six perinatal factors associated with neurodevelopmental referrals in 3-year-old children: infant gender (male), cesarean section, maternal history of psychiatric consultation, EPDS score ≥ 9, lack of social support during pregnancy, and no social assistance post-delivery.

## Introduction

In Japan, child health checkups are conducted at 4 months, 18 months, and 3 years of age, as mandated by the Maternal and Child Health Law, and are free of charge throughout the country^1^. The checkup uptake rate is 94% at 4 months of age, 95.2% at 18 months, and 94.5% at 3 years of age; thus, a high checkup uptake rate is maintained^2^. The checkups include physical measurements, assessment of motor and neural developmental stages, detection of rare diseases, and identification of parents’ concerns regarding childcare. Among these, early detection of neurodevelopmental abnormalities is one of the important objectives of the 3-year-old health checkup because individual differences in children’s development are relatively evident at that age, and the presence or absence of health and medical care support can influence subsequent growth^1^.

Neurodevelopmental disorders, which are characterized by neurological abnormalities, such as cognitive and behavioral characteristics, are often lifelong conditions; however, the causes and pathophysiology are not yet fully understood^3^. Although it is widely accepted that the interaction between genetic and environmental factors creates differences in human cognitive and behavioral characteristics, having a known genetic risk factor does not necessarily guarantee the development of a disease, and this notion has increased the interest in the role of environmental factors^4–6^. Furthermore, because neurodevelopmental disorders develop early in life, there is great interest in the role of the perinatal environment^7–14^. The perinatal environment includes the prenatal environment, such as maternal nutritional status, illness, feelings, stress, and medications, and conditions at birth, such as the delivery method, oxygen and other treatments immediately after birth, and the caregivers’ mental state^15^. Some of these factors, such as maternal nutritional status, feelings, and stress during pregnancy, can be changed with social support, while others, such as maternal illness, the child’s birth weight, delivery method, and postpartum care, are difficult to change.

The most appropriate study design to examine the causal relationship between potential risk factors before and after birth and infant development is a birth cohort study^16^. Although numerous birth cohort studies have been conducted in Europe^17–21^, such studies using data from early pregnancy are very limited in Japan^22–25^. Moreover, there are no reports examining perinatal risk factors for neurodevelopmental problems in 3-year-old children at the screening stage prior to developmental disorder diagnosis using a general population database.

In Kobe City’s maternal and child healthcare program, a unique number is assigned to each fetus when the pregnancy notification form is submitted, and this number can be used to track the child’s health examination results until the child is 3 years old. Therefore, this population-based study aimed to longitudinally investigate perinatal risk factors for neurodevelopmental referrals at 3 years of age in children born and followed up in Kobe, Japan.

## Results

**Figure 1:**
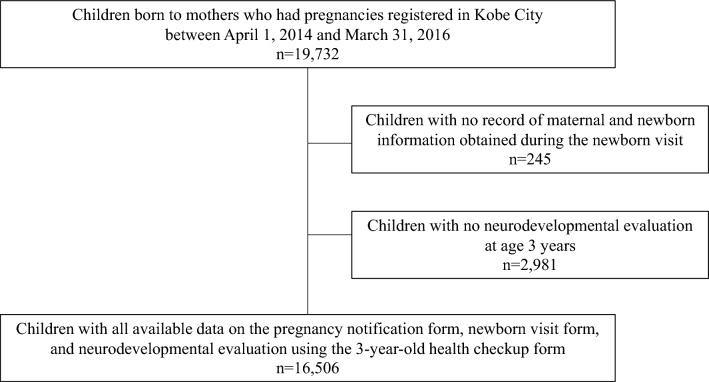
Flowchart of the participants.

Between April 1, 2014, and March 31, 2016, there were 19,732 children whose mothers had pregnancies registered in Kobe City. Of them, 16,506 were included in the study after excluding children who received newborn visits but had no records (n = 245) and whose referrals were not assessed at 3 years of age (n = 2981) (Fig. 1).

**Figure 2:**
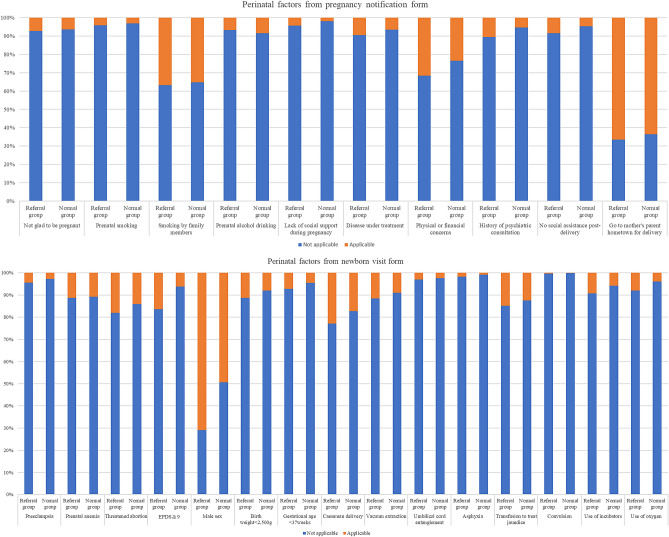
A comparison of the percentage of each factor between the abnormal and normal groups.

Regarding neurodevelopmental referrals at the 3-year-old health checkup, 15,223 and 1283 children were in the normal and referral groups, respectively. Of the 25 factors, univariate analysis showed significant differences in lack of social support during pregnancy, having a disease under treatment, having physical or financial concerns, history of psychiatric consultation, no social assistance post-delivery, maternal preeclampsia, threatened abortion, and Edinburgh Post-natal Depression Scale (EPDS) score ≥ 9, infant gender (male), birth weight < 2500 g, gestational age < 37 weeks, cesarean delivery, asphyxia, use of incubators, and use of oxygen (Fig. 2, Table 1).

**Table 1:** Multivariable logistic regression analysis of perinatal and neonatal factors on neurodevelopment at 3 years of age.

Perinatal factors	Normal group n = 15,223	Referral group n = 1283	Crude OR (99% CI)	P value	Adjusted OR (99% CI)	P value
Factors from pregnancy notification form						
Not glad to be pregnant (chose unexpected and perplexing, not care about anything, or embarrassing)	656/10,269 (6.4%)	69/952 (7.3%)	1.15 (0.82–1.60)	0.30	0.97 (0.67–1.42)	0.86
Prenatal smoking (chose current)	483/15,139 (3.2%)	54/1277 (4.2%)	1.34 (0.92–1.95)	0.05	1.16 (0.70–1.94)	0.44
Smoking by family members living together during pregnancy	5305/15,039 (35.2%)	467/1268 (36.8%)	1.07 (0.92–1.25)	0.27	1.06 (0.87–1.30)	0.43
Prenatal alcohol drinking (chose occasional or daily)	1274/15,119 (8.4%)	85/1274 (6.7%)	0.77 (0.58–1.05)	0.03	1.02 (0.60–1.75)	0.92
**Lack of social support during pregnancy**	**191/10,305 (1.9%)**	**42/962 (4.4%)**	**2.42 (1.55**–**3.78)**	** < 0.001**	**1.99 (1.14**–**3.45)**	**0.001**
**Disease under treatment**	**668/10,289 (6.5%)**	**91/961 (9.5%)**	**1.51 (1.12**–**2.04)**	**0.001**	1.22 (0.86–1.75)	0.15
**Physical or financial concerns**	**2420/10,292 (23.5%)**	**304/961 (31.6%)**	**1.51 (1.25**–**1.82)**	** < 0.001**	1.15 (0.92–1.43)	0.11
**History of psychiatric consultation**	**547/10,301 (5.3%)**	**102/962 (10.6%)**	2.12 **(1.58**–**2.83)**	** < 0.001**	**1.56 (1.10**–**2.22)**	**0.001**
**No social assistance post-delivery**	**485/10,281 (4.7%)**	81/955 (**8.5%)**	**1.87 (1.36**–**2.59)**	** < 0.001**	**1.49 (1.03**–**2.16)**	**0.006**
Go to mother’s parent’s hometown for delivery	6,482/10,199 (63.6%)	633/951 (66.6%)	1.14 (0.95–1.37)	0.07	1.09 (0.89–1.33)	0.27
Factors from newborn visit form						
**Preeclampsia**	**427/15,222 (2.8%)**	**57/1,282 (4.5%)**	**1.61 (1.12**–**2.33)**	**0.002**	1.09 (0.68–1.75)	0.64
Prenatal anemia	1644/15,222 (10.8%)	144/1282 (11.2%)	1.05 (0.83–1.33)	0.64	1.00 (0.74–1.35)	0.99
**Threatened abortion**	**2140/15,222 (14.1%)**	232/1282 **(18.1%)**	**1.35 (1.11**–**1.64)**	** < 0.001**	1.24 (0.96–1.59)	0.03
**EPDS ≥ 9**	**897/14,575 (6.2%)**	**200/1,216 (16.4%)**	3.00 (**2.41**–**3.73)**	** < 0.001**	**1.36 (1.01**–**1.84)**	**0.008**
**Infant gender (Male)**	**7510/15,223 (49.3%)**	**910/1,283 (70.9%)**	**2.51 (2.13**–**2.95)**	** < 0.001**	**2.51 (2.05**–**3.06)**	** < 0.001**
**Birth weight < 2,500 g**	**1207/15,223 (7.9%)**	**144/1,283 (11.2%)**	**1.47 (1.16**–**1.87)**	** < 0.001**	1.30 (0.92–1.84)	0.05
**Gestational age < 37 weeks**	**686/15,082 (4.6%)**	92/1,275 **(7.2%)**	**1.63 (1.22**–**2.19)**	** < 0.001**	1.01 (0.64–1.59)	0.98
**Cesarean delivery**	2636/15,223 **(17.3%)**	**294/1,282 (22.9%)**	**1.42 (1.19**–**1.70)**	** < 0.001**	**1.39 (1.11**–**1.75)**	** < 0.001**
Vacuum extraction	1374/15,221 (9.0%)	148/1282 (11.5%)	1.32 (1.04–1.67)	0.004	1.22 (0.93–1.61)	0.06
Umbilical cord entanglement	372/15,221 (2.44%)	39/1282 (3.04%)	1.25 (0.81–1.94)	0.19	1.31 (0.78–2.18)	0.18
**Asphyxia**	**138/15,221 (0.9%)**	**23/1282 (1.8%)**	**2.00 (1.12**–**3.56)**	**0.004**	1.47 (0.70–3.07)	0.18
Transfusion to treat jaundice	1892/15,221 (12.4%)	191/1282 (14.9%)	1.23 (1.00–1.52)	0.012	0.98 (0.75–1.27)	0.81
Convulsion	18/15,221 (0.1%)	4/1282 (0.3%)	2.64 (0.70–9.97)	0.086	4.45 (0.72–27.74)	0.04
**Use of incubators**	884/15,221 **(5.9%)**	**119/1,282 (9.6%)**	**1.66 (1.28**–**2.16)**	** < 0.001**	0.96 (0.61–1.50)	0.80
**Use of Oxygen**	587/15,221 **(3.9%)**	**102/1,282 (7.9%)**	**2.16 (1.62**–**2.87)**	** < 0.001**	1.57 (0.97–2.55)	0.02

*OR* odds ratio.

Bold text indicates p < 0.01.

A multivariable logistic regression analysis to determine the risk factors for neurodevelopmental referrals at the 3-year-old health checkup showed significant associations in the lack of social support during pregnancy, history of psychiatric consultation, no social assistance post-delivery, EPDS score ≥ 9, infant gender (male), and cesarean delivery. There was no evidence of multicollinearity based on the evaluation of variance inflation factors (all of them were less than 1.8).

## Discussion

This large Japanese cohort study showed that neurodevelopment referrals in 3-year-old children were associated with infant gender, cesarean section, maternal psychological problems before or during pregnancy, the EPDS score, social support during pregnancy, and social assistance post-delivery. Of these, social support during pregnancy and assistance post-delivery were novel factors related to neurodevelopmental referrals in children at age 3 years. We will discuss these six factors associated with neurodevelopmental referrals in 3-year-old children by comparing them with the existing literature.

### Gender differences (male dominance)

Numerous prevalence studies on neurodevelopmental disorders, including attention-deficit/hyperactivity disorder, autism spectrum disorder, and language disorders, are available, and the results have consistently shown a higher prevalence in males^26^. Sex-specific biological factors are involved in the etiology of neurodevelopmental disorders. Possible mechanisms of male gender involvement include genetic risk variants, sex hormones, and observed patterns of sex-specific vulnerability to neurodevelopmental disorders.

### Cesarean section

While previous studies have suggested an association between cesarean section and neurodevelopmental disorders^27–29^, a large Swedish cohort study in 2021 reported no association between cesarean section and abnormal neurological development^30^. This may be, in part, due to the many confounding factors expected to affect the child’s neurological developmental disorders, such as the reason for choosing a cesarean section, whether the cesarean section was an emergency or scheduled, and whether regional or general anesthesia was used.

### Maternal psychological problems before or during pregnancy and EPDS score

Postpartum depression can affect children’s cognitive and behavioral development^31,32^. In Japan, Tachibana et al. reported that mental instability and treatment history for psychiatric illness at 20 weeks gestation are risk factors for postpartum depression^33^. Although our study did not examine the association between maternal mental status during pregnancy and the risk of postpartum depression, mental instability during pregnancy may have affected the neurodevelopment of the child through maternal postpartum depression. In addition, while previous studies evaluated mental status at 20 weeks gestation, our study used pregnancy notification forms. Because pregnancy is typically notified earlier than 20 weeks gestation, the effect of mental status in early pregnancy is a novel finding of this study.

### Social support during pregnancy and social assistance post-delivery

We identified the lack of social support during pregnancy and social assistance post-delivery as maternal loneliness during early pregnancy. No studies have investigated the effects of maternal loneliness during early pregnancy on child development in the general population, and reports focusing on maternal loneliness during pregnancy are limited^34^. Although why these two factors are related to the neurological referrals of 3-year-old children is unclear, associations between pregnancy and postpartum loneliness and postpartum anxiety, depression, low self-efficacy, and stress^34–36^ have been reported, and the association between postpartum anxiety, depression and stress and neurological developmental disorders of the child has also been reported^31,32,37^. Thus, previous reports have shown a relationship between maternal loneliness during child-rearing and postpartum stress, depression, and anxiety disorders, as well as a relationship between postpartum stress, depression, anxiety disorders, and neurological developmental disorders in children. However, our study is the first to clarify the relationship between maternal loneliness in early pregnancy and neurological developmental prognosis at 3 years of age. The importance of our results lies in elucidating the need to identify maternal loneliness in the early stages of pregnancy. Providing social support to pregnant women who need support early in their pregnancy, thereby alleviating their loneliness, may improve neurodevelopment of the newborn.

The results of the present study diverged from many previous reports, as low birth weight, preterm birth^10,38^, smoking^39,40^, and alcohol consumption^41^ were not independent perinatal factors for neurodevelopment referrals. We categorized gestation weeks and birth weight into two groups: < 37 weeks and ≥ 37 weeks and < 2500 g and ≥ 2500 g, respectively. Neurodevelopmental disorders are more pronounced in very preterm infants^10,38^; however, there are few reports on late preterm infants, and late preterm infants were the overwhelming majority of the patients in the present study. In this study, we considered that developmental delay might have been masked by the definition of preterm and low-birthweight infants. In a birth cohort study conducted in Japan, smoking was reported as a possible risk factor for neurodevelopment in newborns^42^. However, a Nordic birth cohort study reported that smoking was not necessarily a risk factor, although no conclusions were reached^43^. It is well known that smoking is harmful to pregnant women and fetuses. The study results may have been affected by the fact that self-reports were collected during the early stages of pregnancy and did not account for smoking behavior or the number of cigarettes smoked after that point.

The strength of our study was its large longitudinal epidemiological design. Kobe has the seventh largest population in Japan. Our study covered almost all pregnant women and newborns in Kobe, thereby avoiding selection bias. This is the first comprehensive analysis of perinatal factors affecting the neurological developmental referrals of 3-year-old children in Japan and the first report in a general population to show that maternal loneliness during early pregnancy is associated with neuro developmental referrals in children.

The limitations of this study were as follows. First, all information on the pregnancy notification forms was based on the mothers’ self-reports; thus, there might have been third-party interventions that caused the mothers to not report the truth. However, because this was a large epidemiological study, the effect is likely small. Second, the neurodevelopmental referrals defined in this study were not directly related to developmental disorders. Moreover, the neurodevelopmental referrals defined in this study include multiple types and degrees of neurodevelopmental issues. As the 3-year-old checkup was conducted as a screening test by trained pediatricians and public health nurses, developmental disorders were unlikely to be overlooked. Nevertheless, some children with future developmental disorders might be overlooked at 3-year-old checkup, and the rate of overlooked disorders was unknown in this study. In contrast, some children with referrals at this checkup will not have developmental disorders in the future. Although pediatricians evaluated neurodevelopmental referrals without perinatal information, the possibility that the pediatrician referred the patient because of a lack of surrounding support cannot be completely ruled out; this health screening system has also not been tested for interrater reliability. Furthermore, in the present study, variables such as medical complications and emotions were treated equally, although the validity of this approach is unclear. Finally, there were potential residual confounding factors. This study did not include maternal age, economic status, or parents’ educational background, which may have affected children’s neurological development. Since this study primarily conducted exploratory analysis, further investigation is necessary to confirm the link between developmental referrals and maternal loneliness during pregnancy.

Although we have used all available items in the existing pregnancy notification form, such items should be considered for inclusion in future studies.

In conclusion, we found that six perinatal factors— infant gender (male), cesarean section, maternal psychological problems before or during pregnancy, EPDS score ≥ 9, lack of social support during pregnancy, and no social assistance post-delivery—were significantly associated with neurodevelopmental referrals in 3-year-old children in the general Japanese population. Some factors, such as maternal loneliness and psychological problems, could be alleviated by social support. Our findings are expected to aid in providing appropriate support for pregnant women and mothers and improve neurodevelopment of children. Because this was a retrospective study, it is unknown whether interventions for perinatal factors found to be associated with neurological developmental referrals in 3-year-olds will actually improve neurodevelopment of children. To demonstrate the usefulness of interventions, it is necessary to conduct a prospective intervention study for mothers with loneliness during pregnancy.

## Methods

### Study design

This was a retrospective, longitudinal, population-based cohort study that used pregnancy notification forms as well as children’s records of newborn visits and 3-year-old health checkups in Kobe City, Japan. The forms for pregnancy notifications, newborn visits, and 3-year-old health checkups were completed using mark sheets, and the completed information was automatically stored in the Kobe City database as anonymized electronic information. This study was conducted at the Kobe University Graduate School of Medicine by accessing the Kobe City database with the approval of the Kobe City Ethics Committee (approval number: 201915). The study protocol was approved by the Ethical Review Board on Health Care of Kobe City*.* Because this was a retrospective data analysis using only anonymized data, this study was exempt from obtaining individual informed consent; instead, an opt-out statement was displayed on the website of Kobe City based on the Ethical Guidelines for Medical and Biological Research Involving Human Subjects. All study methods were carried out in accordance with the approved guidelines.

### Participants

We included children born in Kobe City with available data on the pregnancy notification form, newborn visit form, and neurodevelopmental evaluation using the 3-year-old health checkup form. First, we identified children born to mothers with pregnancies registered in Kobe City between April 1, 2014, and March 31, 2016. We excluded children who had received neonatal home visit guidance but had no record of maternal and neonatal information obtained at the neonatal visit. Similarly, we excluded children who had received a 3-year-old checkup but did not have 3-year-old referrals recorded.

### Perinatal factors and neurodevelopmental referrals at 3-year-old health checkup

We obtained maternal information on feelings during pregnancy (very glad, unexpected but glad, unexpected and perplexing, do not care about anything, embarrassing), prenatal smoking (none, past, current), smoking by family members living together during pregnancy (yes, no), prenatal alcohol drinking (none, occasional, daily), social support during pregnancy (have someone to talk to during the pregnancy, no), having a disease under treatment (yes, no), having physical or financial concerns (yes, no), having a history of psychiatric consultation (yes, no), social assistance post-delivery (have someone to help after delivery, no), going to the mother’s parent hometown to delivery (yes, no) from the pregnancy notification. We also obtained infant information on the child’s birth weight (< 2500 g, ≥ 2500 g), gestational age (< 37 weeks, ≥ 37 weeks), infant gender (male, female), cesarean delivery, vacuum extraction, umbilical cord entanglement, asphyxia, transfusion to treat jaundice, convulsion, use of an incubator and use of oxygen, and maternal information on preeclampsia, prenatal anemia, threatened abortion, and EPDS (rated on a scale of 0 to 30, risk of postpartum depression with a score ≥ 9) from the newborn visit form. All 25 perinatal factors obtained as information were used as potential factors. Each item was collected in a questionnaire form, and the public health nurses also confirmed the information in the interview. These 25 factors were originally prepared for the health checkup for 3-year-old children in Kobe City. Although these factors have not been validated, previous studies have used these factors^44–46^.

The outcome of this study was neurodevelopmental referrals at the 3-year-old health checkup. The evaluation of the 3-year-old health checkup is divided into physical development, neurodevelopment, nurturing status, auditory, visual, and dental categories. The present study used only the neurodevelopmental item among these assessment items.

Neurodevelopmental referrals were evaluated by a trained pediatrician and a public health nurse after medical examination based on seven categories of the 3-year-old health checkup form: (a) normal, (b) required instructions, (c) required re-checkups, (d) required observations, (e) required detailed examination in a hospital, (f) required medical therapy, and (g) received treatment. Of these, children checked in category (a) were defined as the normal group, and children checked in categories other than (a) were defined as the referral group. All pediatricians were trained based on the health screening guidelines to minimize the differences among raters and evaluated the neurodevelopmental referrals without information regarding the mother and child’s perinatal period.

### Statistical analyses

Fisher’s exact test was used to compare the two groups. Data are presented as numbers (percentages) and odds ratio (99% confidence intervals: CI). Multivariable logistic regression analysis was performed to identify perinatal factors associated with neurodevelopmental referrals in children at 3 years of age. All 25 perinatal factors were included as variables in this analysis. All covariates were examined for multicollinearity using the variance inflation factor. P-values < 0.01 were considered statistically significant.

Statistical analyses were performed using SPSS (version 23.0; IBM Corp., Armonk, NY, USA).

## Data Availability

The data that support the findings of this study are available from Kobe City but restrictions apply to the availability of these data, which were used under license for the current study, and so are not publicly available. Data are, however, available from the corresponding author upon reasonable request and with permission of Kobe City.
